# Functional genomics tool: Gene silencing in *Ixodes scapularis *eggs and nymphs by electroporated dsRNA

**DOI:** 10.1186/1472-6750-10-1

**Published:** 2010-01-14

**Authors:** Shahid Karim, Emily Troiano, Thomas N Mather

**Affiliations:** 1 Department of Biological Sciences, University of Southern Mississippi, Hattiesburg, MS 39406, USA; 2 Center for Vector-Borne Disease, University of Rhode Island, Kingston, RI 02881, USA

## Abstract

**Background:**

Ticks are blood-sucking arthropods responsible for transmitting a wide variety of disease-causing agents, and constitute important public health threats globally. *Ixodes scapularis *is the primary vector of the Lyme disease agent in the eastern and central U.S. RNAi is a mechanism by which gene-specific double-stranded RNA (dsRNA) triggers degradation of homologous mRNA transcripts. Here, we describe an optimized protocol for effectively suppressing gene expression in the egg and nymphal stages of *I. scapularis *by electroporation.

**Results:**

The genes encoding the putative Phospholipase A_2 _(PLA_2_), cytoplasmic Cystatin, Syntaxin-5, β-Actin and Calreticulin were targeted by delivering the dsRNA encoding the specific gene coding regions in the unfed nymphs. Silencing was measured using real time qRT-PCR. Electroporation as a mode of dsRNA delivery appears to be substantially efficient and less traumatic to the tick than dsRNA microinjection in the unfed nymphs. Using Cy3-labeled dsRNA to monitor the movement, electroporated dsRNA entered the nymphs and spread to salivary glands and other tissues. The significant disruption of β-actin and cytoplasmic Cystatin transcripts in tick eggs demonstrate the applicability of this technique. The PLA_2_, cytoplasmic Cystatin, Syntaxin-5, β-Actin and Calreticulin genes were also significantly silenced, suggesting that this method has the potential to introduce dsRNA in eggs and unfed nymphs.

**Conclusions:**

Our study demonstrates that electroporation can be used as a simple dsRNA delivery tool in assessing the functional role of tick genes in the vector-host interactions. This technique represents a novel approach for specific gene suppression in immature stages of ticks.

## Background

RNA interference (RNAi) is emerging as a highly effective tool for specific gene disruption. RNAi is an evolutionarily conserved phenomenon of post-transcriptional gene silencing, which is triggered by the presence of 21-23 nucleotides, double stranded (ds) RNA molecules called short interfering RNAs (siRNAs). In cytoplasm, siRNAs from endogenous or exogenous origins interact with a nuclease-containing multiprotein complex called RISC (RNA-induced silencing complex). The siRNAs bind to RISC and unwind, pair with their complementary target mRNA, and allow the RISC complex to cleave the mRNA strand within the target site. This initial cleavage results in rapid degradation of the mRNA molecule, which prevents its translation into protein [1].

While RNAi is greatly facilitating studies to better understand specific gene function, the biggest challenge in using dsRNA among non-model organisms is delivery. To be effective and induce silencing, the dsRNA must reach the cytoplasm of the target cell. RNAi is becoming a routine gene disruption tool in ticks and other systems where genetic manipulations are not feasible [2]. Exogenous delivery of dsRNA has been developed mainly in invertebrates, such as nematodes [1,3] and insects [4,5]. Artificial feeding is one example of a non-traumatic method for delivering dsRNA that preserves the integrity of the treated organism. However, the precise amount of dsRNA taken up can be difficult to monitor. Microinjection has been more widely used in multiple insect species such as mosquitoes, beetles, honey bees, ticks, grasshoppers and aphids [4-11]. Several delivery systems have been attempted for the direct application of dsRNA to different developmental stages of ticks for inducing RNAi *in vitro *and *in vivo *[12,10,12-16]. The ready accessibility of *I. scapularis *nymphs and the important epidemiological role of this tick stage in natural disease transmission cycles make it the favored model system for experimental disease transmission studies. Recently, the possibility of silencing gene expression by RNAi in tick nymphs by dsRNA injection or capillary tube feeding has been reported, albeit with highly variable results [17,18]. Due to their minute size, delivery of dsRNA to unfed nymphal stage ticks remains challenging.

Electroporation is a powerful transfection technique useful for studying gene expression. Initially developed for transfecting *in vitro *cultured cells [19], the technique was adapted to *ex-vivo*, in-situ and *in vivo *DNA transfection of tissue or whole organisms [20]. The principle application of electroporation is focused on vertebrate tissues and organisms with no previous work having been done using ticks. The studies presented here describe gene silencing in *I. scapularis *nymphs and eggs, and explore electroporation as an alternative dsRNA delivery technique. It may be that delivery of dsRNA by electroporation will trigger an RNAi response, inducing specific silencing of tick genes. Specifically, we examined up-take of dsRNA into unfed nymphal ticks and tick eggs, and used qualitative RT-PCR to measure depletion of specific messenger RNA.

## Results

### Delivery of dsRNA into tick nymphs

**Figure 1:**
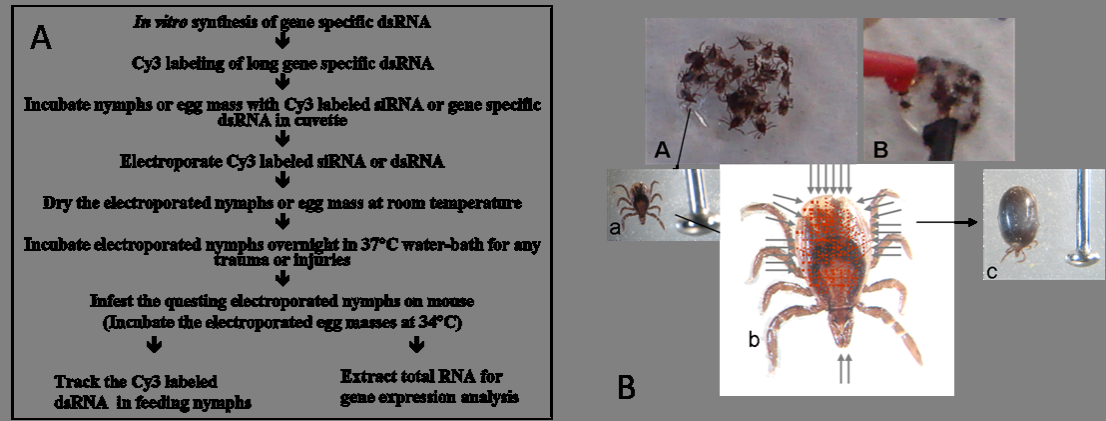
**A) Schematic depicting *in vivo *assay system for gene silencing in tick nymphs and eggs**. All RNAi experiments using candidate tick gene dsRNA followed the same procedure. B: A schematic transdermal delivery of dsRNA by electroporation in tick nymphs. A) Unfed nymphs trapped in dsRNA and, B) electric pulse discharged through electrodes. a) Arrow indicates the size of unfed nymph, b) schematics theoretical influx of labeled long dsRNA in the unfed nymphs and, c) partially feed nymphs after feeding on mouse.

**Figure 2:**
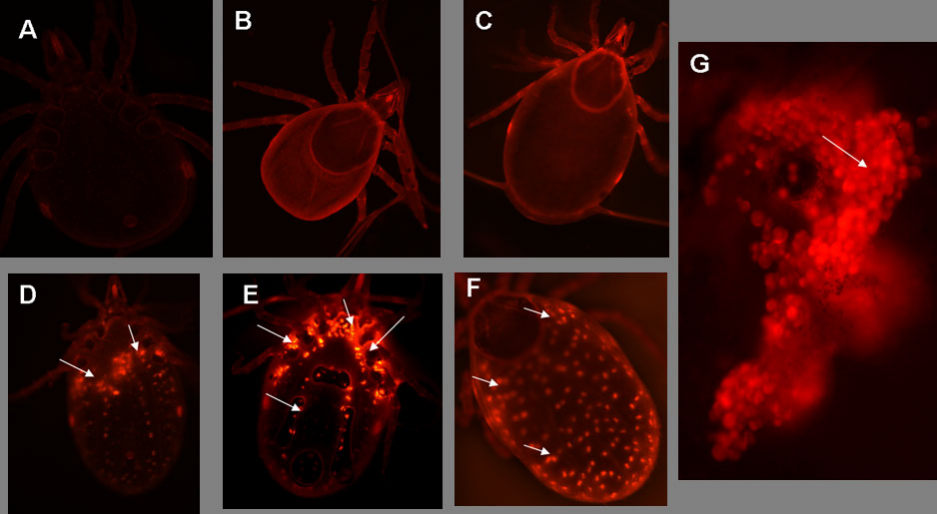
**Visualization of Cy3-labeled dsRNA electroporated into unfed *I. scapularis *nymphs**: A) ventral view, unfed nymph electroporated with Cy3 dye (10×); B) dorsal view of nymphal tick electroporated with Cy3 dye after 24 hrs feeding on mouse (5×); C) dorsal view of nymphal tick electroporated with Cy3 dye after 36 hrs feeding on mouse (10×); D) Ventral view of nymphal tick electroporated with Cy3 labeled GAPDH control siRNA after 48 hrs feeding on mouse (10×); E) Ventral view of 48 hrs fed nymph electroporated with Cy3 labeled Actin dsRNA (10×); F) dorsal view of 48 hrs fed nymph electroporated with Cy3 labeled β-Actin dsRNA; and G) Cy3 labeled Actin dsRNA in the dissected tick salivary gland acini (20×). Arrows indicate the detection of electroporated dsRNA all over the nymphal ticks.

Fluorescein-labeled dsRNA was used to monitor dsRNA uptake following electroporation (Fig. 1). No label was detected in nymphs simply electroporated with Cy3 dye at any time point and this likely reflects the fact that dye is too dispersed in the nymphs (Fig. 2A-C). In nymphs electroporated with Cy3 labeled GAPDH siRNA, small spots of bright fluorescence were detected in salivary glands and midguts (not shown). Intense fluorescent label was also detected in the synganglia area in nymphs (Fig. 2D). All nymphs electroporated with labeled β-Actin dsRNA and then allowed to blood feed for 48 hrs exhibited a strong internal staining pattern (Fig. 2E). Somewhat clustered but distinct internal staining can be seen (Arrows, Fig. 2E) in the area occupied by the salivary glands. At higher magnification, it is possible to see high concentrations of labeled dsRNA associated with these glands (Fig. 2G). In a dorsal view of dsRNA electroporated tick nymphs, the staining has a distinct pattern over internal body structures, suggesting that the label spreads to all tissues (Fig. 2F). When ticks from the 96 hr feeding time point were examined, the staining remained in the salivary glands, and the intensity of label also remained the same over 96 hrs (not shown). In contrast, the intensity of labeling diminished over time in the area occupied by midgut tissues and was no longer detectable by 96 hrs after electroporation. Whether the loss of label is due to degradation, dilution, or its dispersal to undetectable levels throughout the midgut tissues is not known.

To monitor any possible lethal effect of treatment with labeled dsRNA or siRNA or electric pulse to nymphs, all nymphs that attached to the mice after RNAi delivery were considered as alive. Nymphs in all groups were monitored for any lethal phenotype from attachment through detachment (96 hr). No lethal effects of delivered RNAi or electric pulse were observed in any group of nymphs.

### Delivery of labeled dsRNA into tick eggs

**Figure 3:**
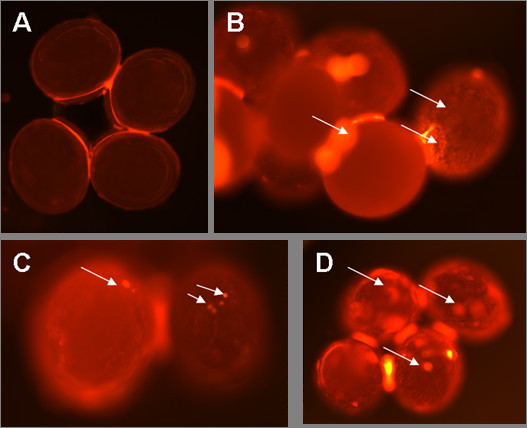
**Visualization of Cy3-labeled dsRNA electroporated into *I. scapularis *eggs**: A) eggs electroporated with Cy3 dye; B) eggs electroporated with Cy3 Actin dsRNA; C) eggs electroporated with Cy3-labeled Actin dsRNA after 2 weeks; D) eggs electroporated with labeled Cy3 GAPDH control siRNAs. Arrows indicate the presence of labeled siRNA or dsRNA in the tick eggs.

In a similar manner, we monitored uptake of fluorescein-labeled tick β-Actin-dsRNA delivered by electroporation into freshly laid *I. scapularis eggs*. Beginning 24 hrs after electroporation and periodically thereafter, electroporated eggs were examined microscopically to track label movement. No label was detected in eggs electroporated with water or Cy3 dye at any time point (Fig. 3A). In contrast, 100% of the eggs electroporated in the presence of Cy3 labeled dsRNA-β-Actin did exhibit an internal staining pattern that was readily detectable from 24 hrs to 2 weeks (Fig. 3B-C). Staining patterns in eggs ranged from diffuse to pinpoint, depending on the focal plane of the eggs (compare Fig. 3C right and left). Electroporation of eggs with Cy3 labeled GAPDH siRNA resulted in more intense staining (Fig. 3D) and suggests greater uptake of this smaller molecule following electroporation. Intensity of staining in eggs did not appear to diminish over time. Ubiquitous distribution of labeled dsRNA or siRNA suggests that it is efficiently spread in the egg.

### Confirmation of gene silencing in the tick eggs and nymphs

Double stranded-RNA-mediated gene silencing in the nymphs using gene specific dsRNA of a putative PLA_2_, cytoplasmic Cystatin, Syntain-5, Calreticulin, and β-Actin was investigated by real time RT-PCR. In one experiment, PLA_2_-dsRNA, irrelevant control *lacZ-dsRNA*, or buffer were delivered by electroporation. Electroporated nymphs were allowed to feed on Balb/C mice. Nymphs attached to mice were removed after 72 hrs of feeding to determine candidate genes expression levels. Ticks electroporated with candidate genes dsRNA showed significantly reduced transcriptional expression when compared with buffer or *lacZ *treatment (Table 1). The *I. scapularis *β-Actin, Cyclophilin A genes were used to normalize levels of mRNA in all samples (Table 1). Experiments using β-Actin-dsRNA also demonstrated transcript depletion in the electroporated nymphs (Table 1). The gene silencing of candidate mRNA due to delivery of dsRNA in unfed nymphs also was confirmed by real time RT-PCR of total RNA from 72 hrs fed nymphs. The result demonstrated that the transcript of all candidate genes were depleted 100% in salivary glands of electroporated nymphs (Table 1).

**Table 1:** Transcriptional silencing of selected genes in *Ixodes scapularis *nymphs

Experimental Group	Gene silencing (72 hrs fed nymphs)	Gene silencing (Salivary glands)
PLA2	95 ± 10.1	97 ± 5.3

β-Actin	91 ± 2.6	98 ± 5.7

Calreticulin	95 ± 4.9	100 ± 0.0

Intracellular Cystatin	100 ± 0.0	100 ± 0.0

Syntaxin 5	90 ± 5.7	100 ± 0.0

**Figure 4:**
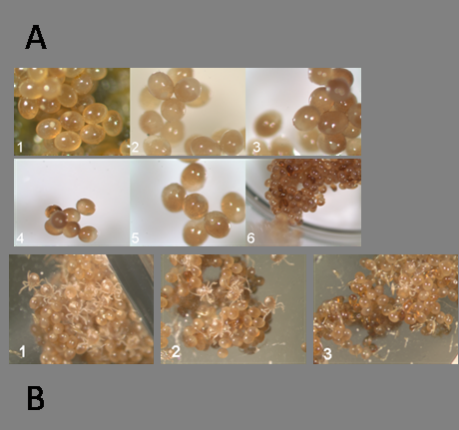
**A) Gene silencing in tick eggs**. Representative *Ixodes scapularis *eggs electroporated, 1) No treatement (3×) 2) water (3×), 3) dsRNA-gfp (3×), 4. dsRNA-*LacZ *(2×), 5-6) dsRNA-β-Actin (3× & 1×). Photographs were taken after 15 days of post-electroporation, B) Representative images taken as larvae started hatching from the electroprated eggs. 1 dsRNA-LacZ (1×), 2. dsRNA-gfp (1×), 3. dsRNA-Actin (1×).

We further determined the efficiency of electroporation for delivering dsRNA to tick eggs by measuring expression of target genes in tick eggs. Eggs electroporated with cytoplasmic Cystatin, β-Actin-dsRNA, buffer and irrelevant *lacZ-dsRNA *and the endogenous expression of target gene was checked using real time RT-PCR one week following electroporation. This experiment revealed reduction in expression of cytoplasmic Cystatin and β-Actin gene transcripts as compared to controls (Table 2). The *I. scapularis *Na^+ ^K^+ ^ATPase gene was used to normalize the levels of mRNA expression in all egg samples. The ability to silence a specific gene in tick eggs suggests that RNAi machinery is active in the tick embryos. We did not see any phenotypic change in eggs electroporated with irrelevant or gene specific dsRNAs (Fig. 4A). Normal larvae hatched from eggs in control and experimental groups (Fig. 4B) but failed to engorge on the host and subsequently died.

**Table 2:** Gene silencing in the *Ixodes scapularis *eggs

Experimental group	Gene Silencing
β-Actin	90 ± 12.5

Intracellular Cystatin	95 ± 5.3

### Impact of electroporation on nymph weight, attachment and feeding success

To investigate any impact of electroporation on tick feeding success, we measured total body weights of nymphs electroporated with buffer, *lacZ-dsRNA*, β-Actin-dsRNA, PLA_2_-dsRNA, cytoplasmic Cystatin-dsRNA, Syntaxin5-dsRNA or calreticulin-dsRNA. Nymphs from each group were weighed individually after detachment from mice, and results were summarized (Table 3). Mean weights of ticks in each treatment group are shown in Table 3. A one-way ANOVA model was marginally significant (***F ***= 2.48, ***df ***= 5, 131, ***P ***= 0.064). Pair-wise comparisons showed significant differences between the Calreticulin, PLA2, Cystatin, syntaxin5 and β-Actin treatments (Duncan's Multiple Range Test, ***P ***< 0.05). A lack of function phenotype was evident in all electroporated nymphs; ~18-52% of the attached nymphs successfully engorged and subsequently dropped off (Table 3). Surprisingly, dsRNA electroporated nymphs remained attached for an average of 6-7 days as compared to control nymphs dropped off from the host in 3 days of attachment (Table 3). Engorged nymphs in control and treated groups successfully molted to the adult stage. The heavier engorged nymphs molted into female adults and light weight nymphs became male adults. Engorged nymphs electroporated withCystatin and Syntaxin-5 dsRNA became male adults. Dropped off nymphs that weighed below 2.5 mg molted into male adult ticks and heavier nymphs became females.

**Table 3:** Weights of ticks treated with test chemicals.

Treatment	Mean weight (mg)	Standard Error	*N (Engorged/total attached)*	*Percent Engorged*	*Average attachment (Days)*
Control	3.436^**ab**^	0.183	55/58	95	3

Calreticulin	4.124^**a**^	0.223	34/65	52	6

β-Actin	3.136^**b**^	0.332	11/62	18	7

PLA_2_	3.500^**ab**^	0.255	32/69	46	6

Cystatin (Cytoplasmic)	2.510^b^	0.215	28/65	43	7

Syntaxin-5	2.113^b^	0.137	25/71	35	7

Means with the same letter do not differ significantly at *P *= 0.05.

## Discussion

The present study was conducted to specifically test the hypothesis that delivery of long dsRNA by electroporation can be used as a potentially high throughput tool for studying functional genomics in epidemiologically important life stages of *I. scapularis*. RNAi has become a powerful experimental tool for studies involving tick-host-pathogen interaction but its utility has been severely limited by the tedious and often traumatic nature of delivering dsRNA to tick nymphs or eggs by microinjection or capillary feeding [17,18]. Therefore, improved delivery methods and optimized protocols for gene silencing in a tick's nymphal or egg stage would make RNAi more widely available to tick biologists and enhance future RNAi applications in tick-host-pathogen and high throughput tick genome research. *I. scapularis *nymphs are responsible for most human transmission of Lyme disease spirochetes, *B. burgdorferi*, and also transform to become either male or female adult ticks, making this stage a favored model system for experimental disease transmission and tick life-cycle studies. In this study, we selected five target genes encoding a biomarker protein (calreticulin), a house-keeping gene (β-Actin), inhibitors of cysteine protease (cytoplasmic Cystatin), exocytotic machinery gene (Syntaxin-5) and a putative Phospholipase A_2_.

In ticks RNAi methodology relies upon the use of long double-stranded RNAs (dsRNA) which, following uptake by the cells, are processed by a Dicer-like enzyme into a pool of 21-23 bp small interfering RNA (siRNAs)[10]. These siRNAs silence endogenous gene expression by triggering cleavage of target mRNAs. One possible explanation for the successes recorded in using long dsRNA in tick gene silencing is the ability to generate siRNAs with high specificity and efficacy. Our studies indicate the relatively similar efficiency of electroporation for delivering either longer dsRNA or siRNA. If introducing longer dsRNA increases the chance of getting a greater number, or more specific siRNAs to gene targets, then it follows that better knockdown efficiency is to be expected.

RNAi is becoming a widely used tool for analyzing the function of specific genes, especially in non-model organisms where systematic recovery of mutants is not feasible as is the case with different developmental stages of ticks. Although injecting dsRNA directly into eggs seems the most effective way to induce an RNAi effect, the number of gene silenced embryos is limited as many embryos do not survive the injection procedure, and it is likely that all individuals are injured in some way by the injection procedure. In species like *Drosophila*, dsRNA injection into embryos sometimes results in a mosaic pattern of knock-down effect suggesting that there is a loss of gene product in some cells and not others [21]. Furthermore, gene silencing frequently kills the embryos, making it difficult to perform functional analysis of these genes at later, post-embryonic stages. Electroporating tick egg masses with Cy3 labeled dsRNA or siRNA appeared to efficiently deliver the desired molecules to tick embryos, and since the label persisted for over 2 weeks (Fig. 3) without any deleterious effect on the egg shell, it seems reasonable to assume that introducing dsRNA in this manner avoided the typical trauma seen in microinjecting eggs. Moreover, qRT-PCR confirmed knock-down of endogenous cytoplasmic Cystatin and Actin mRNA in electroporated eggs suggesting the presence of active RNAi machinery in tick embryos. Recently, injection of dsRNA in the engorged *Boophilus microplus *female was used in efforts to silence gene transcripts in tick eggs [22,23]. Our studies demonstrate that electroporating dsRNA into tick eggs is an efficient, less labor intensive and less traumatic method to newly hatched eggs to target genes exclusively expressing in tick embryos.

Electroporation of Cy3 labeled dsRNA into the unfed pathogen-free nymphs of *I. scapularis *indicated that dsRNA spread throughout the organism. Systemic RNAi has been associated with the Sid-1 protein, a transmembrane protein which enables passive cellular uptake of dsRNA [24,25]. Homologues of other RNAi-associated proteins such as Argonaute, which is the central catalytic component of the RNA-induced silencing complex (RISC) in mammals and arthropods [26], remain to be revealed in *I. scapularis*. The phenomenon of systemic RNAi can be divided into three distinct steps: 1) uptake of dsRNA by cells, 2) processing of dsRNA into small interfering RNAs, and 3) systemic spreading of RNAi effect [27]. Several genes have been identified in *C. elegans *as important for systemic spread but not for the interference itself. Recently, over 20 genes were reportedly necessary for dsRNA uptake in *Drosophila *cells in culture [28,29]. Many of the genes identified as being associated with systemic siRNA spreading also are implicated in endocytsosis, suggesting that this important cellular trans-membrane transport process may play an important role in dsRNA uptake by cells [28,29].

The results reported herein extend those of Nijhof et al. [22], that unprocessed dsRNA in adult ticks not only can be transovarially inherited by the egg stage, but that the RNAi machinery is active in tick eggs. Collectively, our results suggest that although both systemic and transovarial RNAi occurs in ixodid ticks [23,30], gene silencing is reduced as ticks undergo development, probably either as a result of dilution of dsRNA/siRNA or up-regulation of the target gene in the tick tissue. In an elegant study, de la Fuente et al., [31] has shown that RNAi is a very specific process in the ticks.

After nymphs feed to repletion, they molt to either male or female ticks. The heavier nymphs developed into females, the lighter ones became males, and those in between in weight produced both sexes. The same weight-sex relationship was also observed among different tick species; *Ixodes ricinus*, *Ixodes hexagonus*, *Dermacentor variabilis *and *Ixodes scapularis *[32-34]. Unfortunately, we did not check the gene expression of both cystatin and Syntaxin-5 in male tissues, which would shed some light in this aspect. A more fruitful exercise for further study would be to compare single ticks in the group to judge the validity of candidate gene knock down. Clearly, efforts to design better reagents and delivery systems for genetic manipulation of tick phenotypes are still evolving. Delivery of dsRNA and siRNA to ticks by electroporation can be expected to significantly advance RNAi applications over the next few years leading to many exciting discoveries in tick functional genomics and tick-borne disease research.

## Conclusion

RNAi technology has great potential to advance the field of tick biology since it provides a direct method to systematically identify genes involved in variety of pathways implicated in tick feeding or disease transmission to vertebrate hosts. Electroporation of dsRNA into tick eggs and nymphs provides an easy method for the fast characterization of ixodid tick gene function in both stages.

## Methods

Unless otherwise indicated, the protocols followed standard procedures [35], and all the experiments were performed at room temperature (25 ± 1°C). The water used in experiments was of 18 M'Ω quality, produced by a MilliQ apparatus (Millpore, Bedford, MA, USA).

### Ticks

*I.scapularis *ticks were reared using standard methods [36]. Larval ticks were blood fed on hamsters. All unfed nymphs were maintained at 23°C and >90% relative humidity under 14 h light/10 h dark photoperiod before infesting hosts. All studies with animals were performed in accordance with a protocol approved by the Institutional Animal Care and Use Committee at the University of Rhode Island and the University of Southern Mississippi.

### Tissue dissections

Partially fed (72 hrs) pathogen-free nymphs were removed from mice (*Peromyscus leucopus*) using fine-tipped tweezers. Nymph salivary glands and midguts were dissected in ice-cold 100 mM MOPS buffer containing 20 mM ethylene glycol bis-(β-aminoethyl ether)-N, N, N', N'-tetraacetic acid (EGTA), pH 6.8. After dissection, tissues were washed gently in fresh ice-cold buffer. The dissected tissues were stored immediately after dissection in RNAlater (Ambion) prior to extracting total RNA or mRNA.

### Cloning and sequencing of tick genes

Primers were designed based on GENBANK sequences EW812932, DQ066227.1, DN969951, DN972266, DN968449, DQ066345, DN972149 and DN97031 for the *I. scapularis *putative PLA_2_, cytoplasmic Cystatin, Syntaxin-5, β-Actin, α subunit Na^+^-K^+^-ATPase, Cyclophilin A, Cyclophilin G and Calreticulin genes, respectively. Dissected tick tissues stabilized in RNAlater (Ambion) were subjected to RNA extraction using illustra RNAspin Mini RNA isolation kit according to the manufacturers' instructions (GE, USA). All RNA samples were subjected to DNase I treatment as part of RNA extraction protocol. Concentration of total RNA was determined using Nanodrop-100 (Nanodrop Technologies). Total RNA was reverse transcribed using M-MLV (Moloney Murine Leukemia virus) reverse transcriptase according to manufacturers' instructions (Invitrogen). Newly synthesized cDNA was used as a template for PCR reactions using the PCR Supermix (Invitrogen). For each gene, cDNA was PCR amplified using gene specific primers (Table 4).

**Table 4:** Oligonucleotides used to synthesis dsRNA

Target gene	Forward primer (5'-3')	Reverse primer (5'-3')
Na^+^-K^+^-ATPase Alpha subunit (DN968449)	ACGAAACTGCCGAGAGCGACATTA	ATCCTGAGACCTTTGTCCATGCCT

β-Actin (DN972266)	AAACATCCGACATGTGTGACGACGA	TGTGGTGCCAGATCTTCTCCATGT

PLA2 (EW812932)	TTTCCATCGCCACCTGGTATGTCT	ATATTGGCTGCCTGCGTAACGACGA

Calreticulin (DN970315)	TCTTTGCAACGTGGTTTCCTGAGC	TCAGCAGGTTCTTGCCCTTGTAGT

Cystatin-intracellular (DQ066227.1)	TGTTTGCATCGCAGGTCCGT	CACTGGAAGTGCACGATCTCATCT

Syntaxin-5 (DN96995)	ACATTGAGAGCACGATTGTGGAGC	TGCCCAGCATAAATACAGCCCAGA

Cyclophilin G (DN972149)	AGGACCCAAAGTTACCGACAAGGT	TCTCAACCTTGCGTACCACATCCA

Cyclophilin A (DQ066345)	AATAGTGCTCCTCGGTGAAGCCAA	TGCCGAAAGACTCCATCTGCTTGT

All amplifications were performed using a PCR program of 75°C for 3 minutes, 94°C for 2 minutes, 22 cycles of 94°C for 1 minute, 49°C for 1 minute and 72°C for 80 seconds, followed with 10 minutes at 72°C. The PCR products were then cloned into the vector TOPO TA cloning kit. The cloned product was chemically transformed into *E. coli *TOP10 (Invitrogen) as recommended by the manufacturer. Ten colonies were randomly selected and screened for specific gene products by PCR using gene specific primers. Plasmids from positive clones were extracted using QIAquick PCR purification kit (Qiagen). Purified products were quantified spectrophotometrically and sequenced using vector M13 primers. The CEQ8000 Dye terminator Cycle Sequencing with Quick Start kit was used for automated sequencing with the CEQ8000 Genetic Analysis System (Beckman Coulter).

### Synthesis of dsRNA

PCR products of PLA_2_, cytoplasmic Cystatin, Syntaxin-5, Calreticulin and β-Actin genes were joined to the Block-iT T7 TOPO linker. This TOPO linking reaction was used in two PCR reactions with the gene specific and T7 PCR primers to produce sense and anti-sense linear DNA templates. These sense and anti-sense DNA templates were used to generate sense and anti-sense transcripts using BLOCK-iT RNA TOPO transcription kit (Invitrogen, USA). After *in vitro *transcription, each ssRNA was annealed to form dsRNA. An irrelevant dsRNA (*lacZ*) also was synthesized using the pcDNA^α ^1.2/V5-GW/*lacZ *control plasmid and the primers *lacZ *forward 2 (5'-accagaagcggtgccggaaa-3') and reverse 2 (5-ccacagcggatggttcggat-3'). We also used green fluorescent protein gene as irrelevant dsRNA. The size of newly synthesized dsRNA was checked by agarose gel electrophoresis. dsRNAs were quantified spectrophotometrically using a Nanodrop-100. The functional activity of resulting dsRNAs was checked using an *in vitro *gene silencing technique. Briefly, the dissected salivary glands from 20 partially-fed nymphs were incubated with either control *lacZ *dsRNA, tick gene dsRNA or TS/MOPS buffer for 6 hrs at 37°C. After incubation, total RNA was extracted from all samples and gene silencing was checked using RT-PCR.

### Cy3 labeling of β-Actin dsRNA

To be able to track dsRNA during tick feeding, tick β-actin dsRNA was fluorescently labeled using the Cy™3 Silencer siRNA labeling kit (Ambion) with minor modifications to the manufacturer's protocol. β-Actin dsRNA (10 μg) or GAPDH [Glyceraldehyde-3-phosphate dehydrogenase] siRNA (5 μg) were labeled separately by adding Cy3 labeling reagent and incubating for 1 hr at 37°C. GAPDH siRNA was provided in the Ambion Kit. Un-reacted labeling reagent was removed by adding an ethanol precipitation step to the protocol. Briefly, labeled dsRNA/siRNA was precipitated with 0.1 volume of NaCl and 2.5 volumes of 100% ethanol followed by incubation at -20°C for 1 hr. Precipitated, labeled dsRNA was recovered by centrifugation and the pellet was further washed with 70% ethanol. The recovered pellet was dried for 10 minutes at room temperature and re-suspended in nuclease-free water. The concentration of labeled dsRNA and siRNA was determined using a Nanodrop-100 as recommended by the manufacturer.

### Delivery of dsRNA in nymphs by electroporation

To test the delivery of fluorescein-labeled dsRNA, 25 unfed nymphs were immersed in 50 μL of water containing 200 ng Cy3 labeled tick β-actin dsRNA or GAPDH siRNA and were either electroporated [BTX Electro Square Porator ECM 830 (Intracel; 45V, 50ms pulse-width, 10 pulses with 1s intervals between the pulses)] or simply held for 5 min (Fig. 1). After electroporating nymphs with labeled dsRNA, nymphs were removed from the water, dried, and held overnight in plastic vials placed in a covered 37°C water bath incubator before being infested on a naïve Balb/C mouse for blood feeding. Nymphs were removed from the mouse after 24, 48 or 72 hrs of feeding. In another experiment, approximately 150-200 eggs were divided into 6 groups and each group was electroporated with a) water, b) Cy3 dye only, c) labeled GAPDH siRNA, d) labeled β-Actin dsRNA, no treatment. Eggs were kept at 32°C for 96 hrs. Nymphs were cleaned with distilled water after removal from the mouse and visualized under a ZEISS LSM 5 PASCAL Laser Scanning Confocal microscope. Eggs also were visualized the same way using confocal microscopy for the presence of labeled dsRNA.

To test the degree of gene suppression in nymphs, 300 unfed nymphs were divided into five groups of 50 each, and were electroporated with 1 μg of a) β-actin-dsRNA, b) putative PLA_2 _-dsRNA, c) cytoplasmic cystatin, d) Syntaxin-5, e) Calreticulin dsRNA, and f) irrelevant *lacZ *dsRNA in each group. After electroporation, ticks were held overnight at 37°C under high humidity to observe survival. Surviving nymphs were infested on 8 naïve mice and given the opportunity to blood feed till drop off. Partially (72 hrs) fed nymphs were used to extract total RNA for endogenous gene expression. Dropped off engorged nymphs were weighed individually and kept under lab conditions to molt into male or female adults.

Freshly laid tick eggs (~200 in each group) were divided into four groups and electroporated with a) water, b) 500 ng of irrelevant *lacZ *dsRNA, c) β-Actin dsRNA and d) cytoplasmic cystatin. Eggs were held at 32°C and >95% relative humidity for 1 week before processing for total RNA extraction. Total RNA was extracted from eggs using illustra RNAspin Mini RNA isolation kit to test expression levels of β-Actin and cytoplasmic cystatin genes in all groups.

### Confirmation of gene silencing

Real-time quantitative RT-PCR (RT-qPCR) was performed using the Mx3005P Multiplex Quantitative PCR System and the Brilliant SYBR Green Single-Step QRT-PCR Master Mix Kit (Stratagene, La Jolla, CA) according to the manufacturer's instructions. A standard curve (10° to 10^7 ^copies per reaction) was generated using each candidate gene PCR product as the template. The primer sequences used in all qRT-PCR reactions are listed in Table 5. Reactions (25 μl) contained 10 ng of total RNA and were run under the following conditions: 1 cycle of 50°C for 30 min and 95°C for 15 min, followed by 40 cycles of 95°C for 30 s and 55°C for 30 s. Fluorescence was measured at the end of the 55°C step every cycle. Samples were run in triplicate with no-RT and no-template controls. The copy number of candidate gene mRNA in each sample was determined using the Mx3005P data analysis software based on the standard curve [16].

**Table 5:** Oligonucleotides used to quantify gene transcripts

Gene	Forward Primer (5'-3')	Reverse Primer(5'-3')	Product size (bp)
Cytosplasmic Cystatin	TACTTCATCAAGGTTCGCGTTGGC	TGGTGAGCGGAGAAAGTAACCTCT	92

PLA2	TATTGCAAGGCGCTTCACCGAAAG	AAACACTTCAGCCAGCTTGTGACC	97

Calreticulin	ATCATGCGGATCGTGTGCTTGTTG	TCGCTTCCCTTCTTTGTGGAGTGT	137

Syntaxin-5	GTTTGCGGTGCTCATCGTGTTCTT	ATACAGCCCAGAGCAGTCTTGTCA	93

β-Actin	ACATGCTATCGTGGGTGACGAAGT	TGTGGTGCCAGATCTTCTCCATGT	118

NaK ATPase	TGTTGTTCATCTTGGCCGATGTGC	ATGTCGCTCTCGGCAGTTTCGTAT	121

Cyclophilin A	GCTTCGGTTACAAGGGCAGCAGCATTT	TCCGGTGTGCTTCAGGATGAAGTT	149

## Abbreviations

RNAi: RNA interference; dsRNA: double stranded RNA; TBD: tick borne diseases; PCR: polymerase Chain Reaction; Na K ATPase: Sodium Potassium ATPase; siRNA: small interfering RNA; GAPDH: Glyceraldehyde-3-phosphate dehydrogenase; PLA2: Phospholipase A2; RISC: RNA induced silencing complex.

## Authors' contributions

SK conceived molecular design, organized funding, drafted the manuscript and coordinated the study. ET performed experiments. TNM organized funding, participated in the design and helped to draft the manuscript. All authors read and approved the final manuscript.
